# Visualization of the Effect of Assay Size on the Error Profile of Tumor Mutational Burden Measurement

**DOI:** 10.3390/genes13030432

**Published:** 2022-02-26

**Authors:** Nathanael G. Bailey

**Affiliations:** Department of Pathology, University of Pittsburgh and UPMC, Pittsburgh, PA 15213, USA; baileyng@upmc.edu

**Keywords:** tumor mutation burden, immune checkpoint inhibitors, precision medicine

## Abstract

Tumor mutational burden (TMB) refers to the number of somatic mutations in a tumor per megabase and is a biomarker for response to immune checkpoint inhibitor therapy. Immune checkpoint inhibitors are currently approved for tumors with TMB greater than or equal to 10 mutations/megabase. Many laboratories are currently reporting TMB values based upon targeted resequencing panels with limited genomic coverage. Due to sampling variation, this leads to significant uncertainty in the assay’s TMB result, particularly at relatively low TMB levels near the 10 mutation per megabase therapeutic threshold. In order to allow clinicians and laboratorians to explore this uncertainty, we built a novel web application that allows a user to view the potential error of a TMB result given the sequencing panel size. This application also allows the user to explore the effect of incorporating knowledge of a specific tumor type’s typical TMB distribution on the error profile of the TMB result.

## 1. Introduction

Tumor mutational burden (TMB) is defined by the number of somatic mutations present in a tumor exome. TMB is an emerging therapeutic biomarker that is associated with response to immune checkpoint inhibitors (ICI): tumors with higher TMB exhibit better responses to ICIs. This is thought to be due to increased immunogenicity of tumors with high TMB values due to the accumulation of neoantigens [1]. Thresholds ranging from 10 mutations/megabase (mut/Mb) to 20 mut/Mb have been proposed to dichotomize tumors into TMB-low and TMB-high categories [2,3]. Recently, pembrolizumab was approved by the FDA for use in tumors with TMB values of 10 mut/Mb or greater, irrespective of histology [4,5]; this decision has been the subject of much debate [6,7,8,9].

Although TMB is defined as the exomic mutation count, few tumors currently have whole-exome sequencing performed. Instead, mutations are enumerated in targeted sequencing assays that generally sequence around 1 Mb (approximately 1/30th of the entire exome). The mutation number is then divided by the total Mb sequenced to determine TMB. Importantly, these assays were initially designed primarily to assess for known hotspot mutations in oncogenes and for mutations in tumor suppressor genes, and there is no reason to think that the number of mutations in the genes included in the targeted panel is of any more biologic importance for neoantigen formation and ICI response than are mutations of unsequenced genes. Therefore, the validity of the TMB result given by the assay is directly related to its ability to predict the “true,” unmeasured TMB. This ability is dependent on the sampling error of the assay, which is related to the panel size of the sequencing assay. Specifically, if it is assumed that mutations are essentially independent and identically distributed, then the assay’s mutation count is distributed binomially given the number of bases sequenced and the underlying probability of mutation at each base. It stands to reason that sequencing more bases and identifying more mutations improves the error profile of a TMB assay, and it has been well recognized that TMB accuracy is dependent upon the assay panel size [3,10,11,12,13,14,15]. However, the implication of this uncertainty for an individual patient’s TMB result may be difficult for a clinician to visualize. We set out to make an application that displays TMB measurement uncertainty given user-defined inputs of a TMB value and the panel size of the sequencing assay.

## 2. Materials and Methods

An R Shiny web application was written that accepts a target TMB value and the assay size in megabases as user inputs. Based on the entered assay size, the application calculates the assay-specific number of mutations that would need to be detected to most closely generate the entered target TMB value. The probability mass function of the mutation count given the panel size and the underlying true mutation frequency is then modeled with the binomial distribution: *mutation count* ∼ Binomial (*bases sequenced, probability of mutation at each base*).

The probability distribution of the true TMB value given the calculated assay-specific mutation count is determined using the dbinom R function, the user-selected size of the assay, and a vector of “true” mutational probabilities equivalent to 0–200 mut/Mb, in increments of 0.1. The resulting vector of probabilities given a TMB assay result is then normalized and plotted as the likelihood distribution, and it is next sampled to generate information such as the central 90% confidence interval and the likelihood that the “true” TMB value is greater than 10 mut/Mb.

The application additionally draws on publicly available mutational data for patients treated with ICI from cBioportal [16,17] and previously reported by Samstein et al. [18]. This dataset contains TMB values for 1661 patients with various tumor histologies. The user may elect to display histology-specific TMB deciles to visualize the uncertainty of a TMB percentile assignment to an individual case. The R function bkde is used to generate an empiric binned kernel density estimate based on this public mutational data to produce histology-specific TMB prior distributions. At the user’s discretion, the app can incorporate this prior information regarding histology-specific TMB distributions to determine the impact of this knowledge on the measured TMB result using Bayes’s theorem, plotting the prior distribution, the likelihood distribution, and the posterior distribution that takes the prior histology-specific TMB distribution information into account.

The application was built with R v.4.1.0 and packages shiny v.1.7.1 and KernSmooth v.2.23-20. A version of the R script suitable for running on a local R installation using RStudio is contained in the Supplemental Files. The working app is available at https://pathology.shinyapps.io/tmbapp/ (accessed on 7 February 2022).

## 3. Results

### 3.1. Illustration of the Application Interface

**Figure 1:**
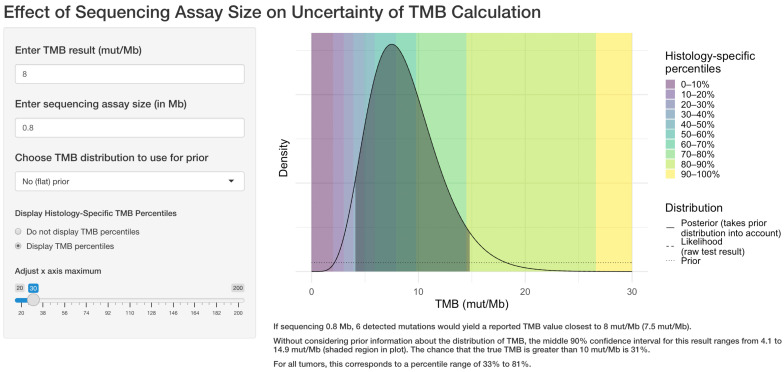
Example of the web application interface. The user selects the target TMB result, the sequencing assay size in megabases, whether to use prior information regarding the TMB distribution for the tumor type, and whether to display TMB percentile information. The application then displays the central 90% confidence interval for the true TMB result and calculates the likelihood that the true result is greater than 10 mutations per megabase.

The user selects a target TMB value, the size of the sequencing assay, and whether to use an informative prior distribution. The application displays the density function of the TMB result, along with the middle 90% confidence interval (CI), the corresponding percentile range, and the probability that the true TMB result is >10 mut/Mb as seen in Figure 1.

### 3.2. Effect of Assay Size on Uncertainty

**Figure 2:**
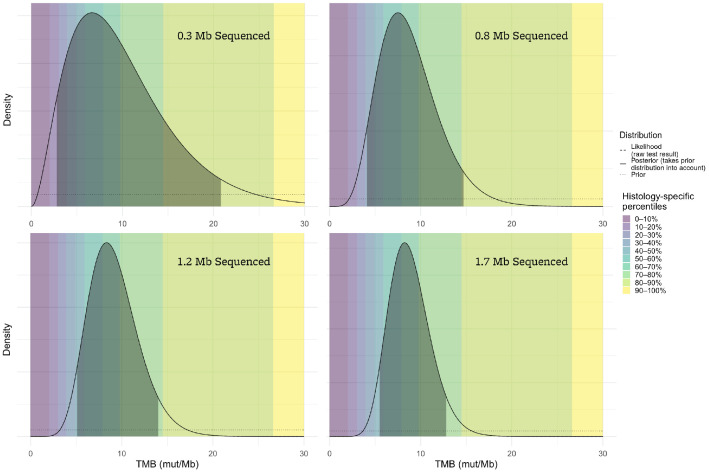
The accuracy of the TMB result is highly dependent upon the number of megabases sequenced. If very few megabases are sequenced (top left panel), the range of plausible true TMB values (indicated by the shaded areas of the plots) given the assay result is very broad, while results from larger sequencing assays (such as the bottom right) are more reliable.

Figure 2 illustrates the significant effect of assay size on the confidence interval and corresponding percentile range for a TMB result of 8 mut/Mb.

### 3.3. Significant Assay Uncertainty at Low TMB Values

**Figure 3:**
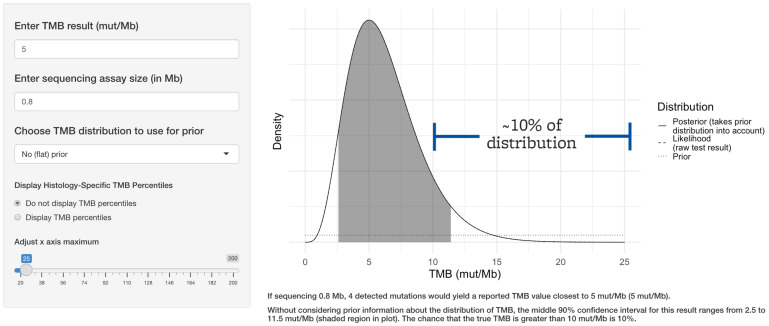
The threshold for clinical response to immune checkpoint inhibitor therapy is 10 mut/Mb based on TMB alone. FDA-approved companion diagnostic TMB assays sequence approximately 0.8 Mb. Even a common TMB-low value such as 5 mut/Mb generated by such an assay would indicate a true TMB value of greater than 10 mut/Mb approximately 10% of the time.

If approximately 0.8 Mb is sequenced, such as in the FDA-approved companion diagnostic test for pembrolizumab, a TMB-low result of 5 mut/Mb could actually reflect a true TMB value of >10 mut/Mb approximately 10% of the time, as seen in Figure 3.

### 3.4. Effect of Incorporating Knowledge of Prior Distribution

If significantly less than 1 Mb is sequenced, the confidence interval of TMB results is highly dependent upon the tumor-specific prior distribution.

**Figure 4:**
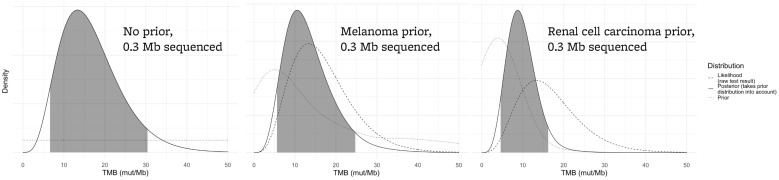
For a small assay sequencing 0.3 Mb, the confidence interval of a TMB result is affected by the tumor type that is being sequenced. Each plot assumes an assay result of 12 mut/Mb. The left panel demonstrates the likely true TMB result (gray area) without taking prior knowledge of tumor TMB distributions into account. The true TMB result will likely be higher in a tumor type that tends to have higher TMB values (such as melanoma, center plot) versus a tumor type that tends to have lower TMB values (renal cell carcinoma, right plot).

If only 0.3 Mb is sequenced, a TMB value of 12 mut/Mb would suggest that there is an 81% chance that the true TMB is greater than 10 mut/Mb, assuming a flat prior (Figure 4, left panel). However, most tumors have TMB values less than 10 mut/Mb, and incorporating that a priori information will tend to decrease the range of plausible values for the “true” TMB result. Different tumors have different TMB distributions, and this effect can greatly impact the implications of a TMB result if small sequencing panels such as this are used. For example, with an assay that is 0.3 Mb in size, if the sample is melanoma (with a high pretest probability of increased TMB), the probability that an assay result of 12 mut/Mb indicates a true TMB result of >10 mut/Mb is 68% (Figure 4, center). However, if the sample is a renal cell carcinoma (where TMB is expected to be low), the probability is only 43% (Figure 4, right panel).

**Figure 5:**
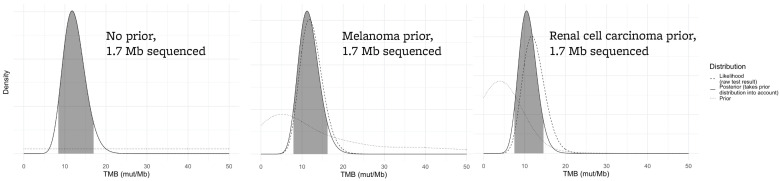
For a larger assay sequencing 1.7 Mb, the effect of the prior distribution on the “true” TMB result is less marked. All panels assume an assay-generated TMB value of 12 mut/Mb.

As demonstrated in Figure 5, if 1.7 Mb is sequenced, the posterior distribution of a TMB result of 12 mut/Mb is far less dependent on the prior because the test result itself is much more informative (likelihood that the true TMB is greater than 10 mut/Mb: flat prior 79%, melanoma 73%, renal cell carcinoma 63%).

### 3.5. Very High TMB Values Are Less Problematic

**Figure 6:**
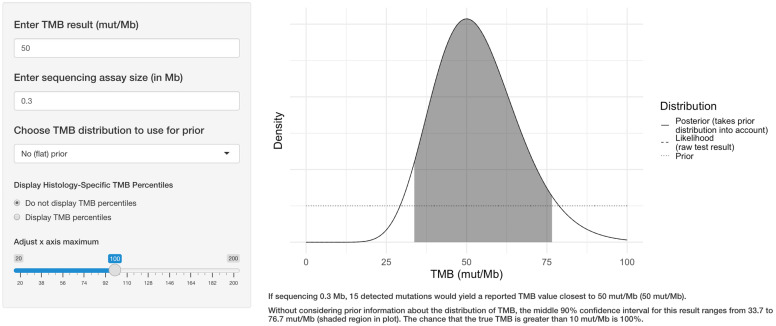
If many mutations are identified far from the 10 mut/Mb threshold, even very small panels can reliably establish that a tumor is TMB-high with confidence.

Although significant uncertainty remains with regard to the true TMB value, even small targeted sequencing panels are adequate to classify a tumor as TMB-high if many mutations are identified, as seen in Figure 6.

## 4. Discussion

The web application provides a user-friendly and convenient way to explore the uncertainty of a TMB result due to sampling error. There are several limitations of this application. Sources of TMB measurement error other than those derived from sampling such as variant allele fraction thresholds, distinction between germline and somatic variants (which may be particularly challenging for patients from under-represented groups [19]), and potential targeted panel bias toward hotspot mutations are not reflected in the output of the application. These additional sources of error are likely substantial when using small panels, where each incremental mutation identified has a large effect on the TMB calculation. This application also makes some simplifying assumptions regarding the distribution of mutations through the exome, as tumors contain mutational signatures reflecting their underlying mutational processes, and the distributions of mutations throughout the exome are not entirely random [20]. These sources of assay uncertainty are not reflected in the simple binomial model used in this application, and the output of the application should be considered a first approximation of the assay error.

Clinicians are familiar with the concept that some laboratory results need to be interpreted in conjunction with the pre-test probability of a condition: for many assays, clinicians are trained to consider an assay’s sensitivity and specificity in light of a condition’s prevalence to determine the more clinically meaningful positive and negative predictive values for the assay result. Ideally, analytical laboratory assays would be sufficiently robust such that the pre-test prior distribution of possible results is relatively unimportant when interpreting the results of the assay, but this is not the case with TMB results as currently measured. The prior distribution of TMB values for a given tumor type can significantly impact the plausible “true” TMB value, just as the prevalence of a disease can markedly impact the predictive value of an assay with a given sensitivity and specificity. Incorporating prior knowledge about the histology-specific TMB distribution can lead to a more accurate estimation of TMB, particularly if very small sequencing panels are used. Since most tumors have TMB values <10 mut/Mb, incorporating this knowledge typically decreases the plausible TMB range. For example, a TMB-high result is more likely to be “true” if a melanoma is being sequenced than if a renal cell carcinoma is being sequenced, unless the assay is of sufficient size to mitigate the uncertainty due to sampling error or unless a sufficiently high number of mutations are identified.

Studies have shown good correlation between TMB determined by targeted sequencing panels and ICI response [4,18], but sampling errors as modeled in this application would normalize across a large cohort of patients; showing that an assay correlates with an outcome using cohort-level metrics has limited implications for an individual patient’s result if it cannot be accurately measured. In a cohort of patients with TMB measured by small panels, some patients’ TMB values will be overestimated and others will be underestimated, still leading to an positive overall association between TMB and ICI response, as the measurement errors will tend to cancel one another out and the overall trend will remain. However, in routine clinical practice, the question is not whether an assay’s TMB results correlate with ICI response across a cohort of patients; rather, it is “can the assay accurately determine the TMB-high/low status and therefore therapy *for this individual patient?*” Unfortunately, currently used small sequencing panels cannot definitively answer this question for the great majority of patient tumors with TMB values that are below or near the TMB-high threshold of 10 mut/Mb, as has been recognized in ongoing TMB standardization efforts [14]. TMB is likely a predictor for ICI response as a continuous variable, reflecting increasing neoantigen density in a tumor. While any threshold used for dichotomization of a continuous variable is intrinsically arbitrary and difficult to justify [21], the current FDA-approved threshold for TMB is especially problematic, as a patient’s TMB cannot be measured with confidence near the 10 mut/Mb threshold with many currently used assays. However, for highly mutated tumors, such as those with DNA mismatch repair deficiency or *POLE/POLD1* mutations (also populations where the benefit of ICI is most clear [9,22,23,24]), the clinical performance of assays with small panel sizes may be clinically adequate, as the plausible interval for the true TMB value will be unlikely to contain the TMB-high threshold in spite of the increasing variation in the actual numerical TMB result generated by the assay [14].

## 5. Conclusions

The application provides a convenient way to approximate the error intrinsic to TMB measurement by targeted resequencing approaches. Laboratories reporting TMB should consider acknowledging the uncertainty of TMB results in their reports, particularly near clinically relevant thresholds. Assays that sequence significantly less than 1 Mb should not be used to determine TMB. Ongoing standardization efforts will remain important, along with additional studies to better determine biomarkers that are most predictive of ICI response.

## Data Availability

The R code for the web application is available as a Supplemental File.

## Supplementary Materials

The following supporting information can be downloaded at: https://www.mdpi.com/article/10.3390/genes13030432/s1, File: app.R, which provides code sufficient to run the application on a local R installation.

## Abbreviations

The following abbreviations are used in this manuscript:

TMB	Tumor mutational burden
ICI	Immune checkpoint inhibitor
mut/Mb	Mutations per megabase
